# Male guanine-rich RNA sequence binding factor 1 knockout mice (*Grsf1*^*−/−*^*)* gain less body weight during adolescence and adulthood

**DOI:** 10.1186/s13578-022-00922-3

**Published:** 2022-12-09

**Authors:** Bernhard Dumoulin, Dagmar Heydeck, Desiree Jähn, Moritz Lassé, Sajad Sofi, Christoph Ufer, Hartmut Kuhn

**Affiliations:** 1 Department of Biochemistry, Charité - University Medicine Berlin, Corporate Member of Free University Berlin, Humboldt University Berlin and Berlin Institute of Health, Charitéplatz 1, 10117 Berlin, Germany; 2 Present Address: Department of Medicine, University Medical Center Hamburg-Eppendorf, Hamburg, Germany; 3 Present Address: Department of Biology, University of York, York, YO10 5DD UK

**Keywords:** Gene expression regulation, Lipoxygenase, RNA binding proteins, Redox homeostasis, Embryogenesis, Hematopoiesis

## Abstract

The guanine-rich RNA sequence binding factor 1 (GRSF1) is an RNA-binding protein of the heterogenous nuclear ribonucleoprotein H/F (hnRNP H/F) family that binds to guanine-rich RNA sequences forming G-quadruplex structures. In mice and humans there are single copy *GRSF1* genes, but multiple transcripts have been reported. GRSF1 has been implicated in a number of physiological processes (e.g. embryogenesis, erythropoiesis, redox homeostasis, RNA metabolism) but also in the pathogenesis of viral infections and hyperproliferative diseases. These postulated biological functions of GRSF1 originate from in vitro studies rather than complex in vivo systems. To assess the in vivo relevance of these findings, we created systemic *Grsf1*^−/−^ knockout mice lacking exons 4 and 5 of the *Grsf1* gene and compared the basic functional characteristics of these animals with those of wildtype controls. We found that *Grsf1*-deficient mice are viable, reproduce normally and have fully functional hematopoietic systems. Up to an age of 15 weeks they develop normally but when male individuals grow older, they gain significantly less body weight than wildtype controls in a gender-specific manner. Profiling *Grsf1* mRNA expression in different mouse tissues we observed high concentrations in testis. Comparison of the testicular transcriptomes of *Grsf1*^−/−^ mice and wildtype controls confirmed near complete knock-out of *Grsf1* but otherwise subtle differences in transcript regulations. Comparative testicular proteome analyses suggested perturbed mitochondrial respiration in *Grsf1*^−/−^ mice which may be related to compromised expression of complex I proteins. Here we present, for the first time, an in vivo complete *Grsf1* knock-out mouse with comprehensive physiological, transcriptomic and proteomic characterization to improve our understanding of the GRSF1 beyond in vitro cell culture models.

**Supplementary Information:**

The online version contains supplementary material available at 10.1186/s13578-022-00922-3.

## Introduction

RNA binding proteins (RBP) are among the most abundant proteins in the cell [1]. They are ubiquitously expressed and have been implicated in transcriptional as well as post-transcriptional regulation of gene expression [1]. The heterogenous nuclear ribonucleoprotein (hnRNP) H/F family of RBP is characterized by the presence of at least two RNA recognition motifs (RRM) [2]. The hnRNP H/F proteins regulate different cellular processes such as nucleo-cytoplasmic traffic, splicing, polyadenylation and 3’-end processing of pre-mRNA [3–7]. GRSF1 is a unique member of the hnRNP H/F family since it executes some of its functions in mitochondria.

The human *GRSF1* gene lies on the long arm of chromosome 4 (4q13.3) and spans 24 kbp with 9 exons and 8 introns [8]. The corresponding mouse ortholog has been mapped to a syntenic region of chromosome 5 (88,659,448–88,676,171 bp) [9]. The *GRSF1* gene is highly conserved in lower and higher mammals and also present in *Xenopus tropicalis* (clawed frog), *Danio rerio* (zebrafish) and *Gallus gallus* (chicken) [8]. Moreover, *GRSF1* like genes have been detected in some viruses, fungi, plants, insects and worms including *Drosophila melanogaster* and *Caenorhabditis elegans* [8]. Several GRSF1 isoproteins originate from the human GRSF1 gene. These differ from each other in respect to their N-terminal sequences [10]. The two dominant isoproteins are isoform 1, full length GRSF1 (UniProt ID: Q12849, isoform 1) and isoform 2 (UniProt ID: A0A024RD99) which lacks the N-terminal Alanine-rich domain [10]. The Ala-rich domain has been predicted to include a mitochondrial targeting signal sequence, therefore isoform 1 can be found in mitochondria whereas isoform 2 is only found in the cytoplasm [11, 12]. Moreover, the Ala-rich domain seems to be required for protein–protein interaction [13]. Besides the Ala-rich domain, full length GRSF1 is comprised of three RNA binding domains (RBD) and an acidic amino acid-rich domain [14]. The latter is hypothesized to regulate RNA binding affinity [10]. The three RBD are denoted quasi-RNA recognition motifs (qRRM), their structure is hypothesized to be similar to RRM and consists of four anti-parallel β-strands that are interrupted by two α-helices to form a βαββαβ-topology [15]. However, despite their similar structure RRMs and qRRMs differ in their RNA binding behavior. Whereas RRMs bind RNA via specific hydrogen bonds with amino acid residues of their central β sheets, qRRMs bind RNA substrates with amino acid residues localized in the loop regions interconnecting the central β sheets of the βαββαβ motif [15].

GRSF1 specifically interacts with Guanine-rich stretches of RNA which may potentially fold into G-quadruplex structures [16]. These secondary structures are stabilized by additional Hogsteen base pairing which induces the formation of four-stranded structures and stacked guanine quartets [17]. They are preferentially located in the 3ʹ- or 5ʹ untranslated regions (UTR) of mRNA. RNA G-quadruplexes have been shown to act as regulatory elements in splicing, polyadenylation and translation [18]. There are three well characterized RNA substrates of GRSF1, *unconventional SNARE in the ER 1* (*Use1*) [19]*, glutathione peroxidase 4* (*GPx4*, [20]) and the *viral Influenza virus nucleoprotein* RNA [21]. GRSF1 has been shown to bind to Guanine-rich sequences in the 5’ UTR of these mRNAs. *In sililco* structure modeling of these mRNAs suggested a high probability that the Guanine-rich sequences of the 5’ UTR fold into G quadruplexes [14]. More recently, two new RNA substrates of GRSF1 have been identified, the *neuroblastoma RAS viral oncogene homolog* (*NRAS*, [22]) and *Mito3* a mitochondrial L strand transcript [23]. Both of these RNAs form G quadruplex structures that were bound by GRSF1 [22, 23]. Interestingly, GRSF1 seems to unwind the G quadruplex structure of *Mito3* and allow its degradation by the mitochondrial degradosome [23]. However, given the low amounts of characterized RNA substrates of GRSF1 it remains to be elucidated whether G quadruplex formation is a necessary precondition for GRSF1 binding.

GRSF1 has been implicated in a number of pathophysiological as well as physiological processes. For example, GRSF1 has been shown to positively regulate the translation of the *Use1* mRNA [19]. In an ex vivo experimental setup using an siRNA mediated knockdown of *Grsf1*, regulation of *Use1* translation was shown to be important for the mTOR dependent amplification of the erythroblast compartment in erythropoiesis [19]. Similarly, GRSF1 has been shown to positively regulate *Gpx4* expression by binding to an AGGGA motif in its 5’ UTR [20]. Using siRNA mediated knockdown of *Grsf1* in an in vitro murine embryogenesis model [20], it was shown that deficiency of GRSF1 led to marked increase in lipid peroxidation, superoxide levels, DNA damage and p53 activation [20]. Moreover, GRSF1-deficient embryos displayed disturbed brain development, highlighting a role of GRSF1 in murine redox homeostasis and embryogenesis [20]. In mitochondria, GRSF1 has been show to play a role in RNA metabolism. GRSF1 colocalizes with mitochondrial RNA granules (MRG), responsible for post-transcriptional modifications of mitochondrial (mt) RNA, translation and miRNA degradation [11]. In these MRG GRSF1 is hypothesized to be involved in tRNA maturation due to GRSF1 depletion leading to a decrease in mature mt-RNA species [11]. Furthermore, GRSF1 seems to be important for mitochondrial ribosomal biogenesis [11] with absence of GRSF1 leading to decreased expression of mitochondrial proteins of complex II and IV of the respiratory chain [12]. Interestingly, a current study reported significantly impaired catalytic activity of complexes I and IV in GRSF1 deficient HEK293 cells [24]. Most recently, GRSF1 was also shown to be involved in mitochondrial RNA degradation: GRSF1 colocalizes with the mitochondrial degradosome and deficiency of GRSF1 led to an accumulation of different mitochondrial transcripts [23].

Together, these postulated biological roles of GRSF1 have all been derived using in vitro or ex vivo approaches. Only recently Driscoll et al*.* generated a floxed *Grsf1* mouse line and crossed these animals with *Myogenic factor 5 (Myf5) -Cre* mice [25]. In these mice the expression of Cre recombinase is controlled by the promoter of the *Myf5* gene, which is almost exclusively expressed in skeletal muscle tissue. Therefore, expression of *Grsf1* is abrogated in skeletal muscle tissue of these mice. Interestingly, the authors found that at higher age these Grsf1-deficient animals showed a weakened muscle endurance [25]. However, so far characterization of general *Grsf1* knockout mice has not been published [8].

In the present study, we created a floxed *Grsf1* mouse line and crossed these animals with *CMV-Cre* deleter mice. Expression of *Grsf1* is therefore deleted in all tissues including germ line cells. We found that these mice are viable, reproduce normally and display no major phenotypic alterations when compared with wildtype C57Bl/6 mice. Comparison of differential blood counts of wildtype and our *Grsf1*^−/−^ mice revealed no significant differences. Interestingly, male *Grsf1*^−/−^ mice gain significantly less body weight when aging starting at an age of about 15 weeks postpartum. Since GRSF1 is expressed at high levels in testis we carried out combined transcriptome and proteome analyses and detected subtle differences between the two genotypes. On the proteome level, we found reduced expression of proteins involved in complex I of the mitochondrial electron transfer chain, consistent with the previously described role of GRSF1 in mitochondrial respiration [11, 12, 26].

## Materials and methods

### Chemicals and devices

**Fig. 1:**
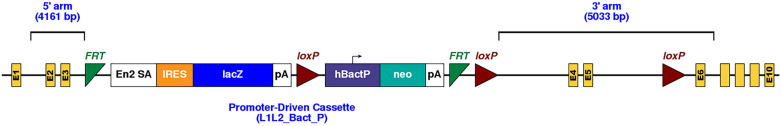
Functional silencing of the *Grsf1* gene [C57BL/6J Grsf1tm1a(EUCOMM)Wtsi]. An artificial knockout exon was cloned between two FRT recognition sites (flipase recognition target) and this construct was used for transfection of mouse embryonic stem cells. This exon involves a splice acceptor side (En2 SA), an internal ribosomal insertion site (IRES), an open reading frame encoding for the beta-galactosidase gene (LacZ) and a polyadenylation sequence (PA). This sequence is followed by a *loxP* sequence and a neomycin resistance cassette consisting of the human beta-actin (hBactP) promoter and a downstream polyadenylation signal (PA). Immediately downstream of the PA signal second FRT sequence was included. Finally, the construct involves two unidirectional *loxP* sequences flanking exons 4 and 5. Stem cells that have incorporated this construct by homologous recombination were used for blastocyst injection and chimeric mice were bred. Crossing individuals containing this additional exon with FLT delete mice removes the sequences between the FRT signals but maintains the *loxP* flanked exons 4 and 5 (*Grsf1*-floxed mice). When these *Grsf1*-floxed mice were crossed with Cre-deleter mice, in which expression of the Cre recombinase is governed by unspecific or tissue specific promoters, tissue specific inactivation of the *Grsf1* gene can be achieved. To induce systemic *Grsf1* knockout in all cells and tissues we employed CMV-Cre mice (B6.C-Tg(CMV-cre)1Cgn/J) and this strategy truncated the *Grsf1* gene in all cells and tissues removing exons E4 and E5

The chemicals used for the different experiments were obtained from the following sources: Phosphate buffered saline without calcium and magnesium (PBS) from PAN Biotech (Aidenbach, Germany); nitrocellulose blotting membrane from Serva Electrophoresis GmbH (Heidelberg, Germany); EDTA Merck (Darmstadt, Germany); acetonitrile (HPLC grade for peptide LC–MS) from Merck (Darmstadt, Germany); trypsin from Serva (Heidelberg, Germany). The knockout construct (Fig. 1) was kindly provided free of charge by the European Conditional Mouse Mutagenesis (EUCOMM) program. The origins of other chemicals and devices used in this study are specified when the different analytical and preparative methods are described.

### Genotyping

Mouse tail or ear biopsies were incubated in 1.5 ml tubes for 30 min in 40 µl lysis buffer (25 mM NaOH, 0.2 mM EDTA, pH 12) at 95 °C under mild agitation. Precipitate was spun down at 15,000 rpm for 30 s, 40 µl of neutralizing buffer (40 mM Tris/HCl pH 5.0) were added, the liquid was recovered and the sample was stored on ice until further use or was stored at − 20 °C. The following primer combinations were used for genotyping. Wildtype mice: *Grsf1* forward, *5ʹ*-TCA GTG AAG AAC GCT CTT GTT GGC-*3ʹ* and 24140 down: *5ʹ*-GAA CTT CTT GGA TTC TGG CTC ACA-*3ʹ* (556 bp amplification product). *Grsf1*^*−/−*^ mice: *Grsf1* 5ʹarm *5ʹ*-TTT GTG TGG TAG GGT TCA CGT GGG-*3ʹ* and 24140 down *5ʹ*-GAA CTT CTT GGA TTC TGG CTC ACA-*3ʹ* (682 bp amplification product). The PCR reaction mixture containing 2 µl genomic DNA, 0.4 µl dNTPs (10 mM each), 1 µl Primer *Grsf1* (5 µM), 2 µl Primer 24140do (5 µM), 1 µl Primer *Grsf1* 5'arm (5 µM), 4 µl Phusion HF Buffer, 0.6 µl DMSO, 0.2 µl Phusion HS II DNA Polymerase (Thermofisher Scientific, Dreieich, Germany) and 8.8 µl sterile water was incubated according to the following PCR protocol: initial denaturation 1 min 98 °C; 31 × cycle: 20 s at 98 ℃ denaturation, 30 s 68 ℃ annealing, 30 s 72 ℃ extension; final extension 3 min 72 ℃. PCR products were separated in an 1.5% agarose gel and were visualized on an UV-gel imager.

### RNA extraction

Tissue was disrupted in Lysing matrix D tubes containing 600 μL LBP (NucleoSpin kit) with a FastPrep 24 homogenizer (MP Biomedicals GmbH, Eschwege, Germany; 3 × 40 s). Debris was spun down, the supernatant was recovered and cleared using Qia-shredder columns (Qiagen GmbH, Hilden, Germany). Total RNA was extracted from this eluate using the NucleoSpin RNA Plus mini kit from Macherey–Nagel (Düren, Germany) according to the instructions of the vendor.

### cDNA synthesis

0.5 µg of total RNA were reversely transcribed using the Tetro Reverse Transcriptase kit (Bioline, Luckenwalde, Germany) according to the following protocol: RNA and RNAse free water were mixed in a total volume of 12 µl containing 0.5 µg RNA. 7 µl of reaction mixture (4 µl fivefold Reaction Buffer, 1 µl dNTP’s (10 mM each), 1 µl Oligo dT18 (10 µM), 1 µl RNase Inhibitor (Bioline, Luckenwalde, Germany). 1 µl Tetro reverse transcriptase were added and the samples were incubated for 1 h at 45 ℃. The reaction was terminated by incubating the sample for 5 min at 85 °C.

### qRT-PCR

Amplification standards for mouse *Gapdh* mRNA and mouse *Grsf1* mRNA were cloned into the TOPO 2.1 vector (Invitrogen-Thermofisher Scientific, Dreieich, Germany) and the linearized vector with known ssDNA copy number/μL was used as calibration standard for amplification. qRT-PCR (SensiFastSybr no-Rox Kit; Bioline, distributed by BioCat GmbH Heidelberg) was performed on a Rotor-Gene RG 3000 (Corbett Research Ltd., Saffron Walden, UK) instrument according to the following protocol using 1 µl of cDNA and gene specific primers (mouse *Gapdh* forward 5ʹ-CCA TCA CCA TCT TCC AGG AGC GA-3ʹ, mouse *Gapdh* reverse 5ʹ-GGA TGA CCT TGC CCA CAG CCT TG-3ʹ; mouse *Grsf1* forward 5ʹ-GAA TCC AAA ACT ACC TAC CTG GAA G-3ʹ, mouse *Grsf1* reverse 5ʹ-CAG CTG TAA GGA AGT CCT CTC AG-3ʹ. Initial denaturation step 10 min at 95 ℃; 40 × cycle: denaturation 15 s at 95 ℃, annealing 30 s at 65 ℃, extension 20 s at 72 ℃.

### Transcriptome analysis

To compare the testicular transcriptomes of *Grsf1*^−/−^ mice and wildtype controls (C57BL/6 mice) we extracted total RNA from testis of three male *Grsf1*^−/−^ mice and from four wildtype control animals (C57BL/6 mice) using the NucleoSpin RNA Plus mini kit from Macherey–Nagel (Düren, Germany). The library construction and sequencing and bioinformatic data analysis were performed by Novogene Europe (Cambridge, UK). Briefly, mRNA was purified using poly-T oligo-attached magnetic beads. Sequencing libraries were generated using NEBNext^®^ UltraTM RNA Library Prep Kit for Illumina^®^ (NEB, USA) following manufacturer’s recommendations, and index codes were added to attribute sequences to each sample. The clustering of the index-coded samples was performed on a cBot Cluster Generation System using TruSeq PE Cluster Kit v3-cBot-HS (Illumia) according to the manufacturer’s instructions. After cluster generation, the library preparations were sequenced on an Illumina Novaseq 6000 platform and 150 bp paired-end reads were generated. For the data analysis, raw data (raw reads) in fastq format were first processed. Clean data (clean reads) was obtained by removing reads containing adapters, reads containing N > 10% (N represents base that could not be determined) and low-quality reads from raw data. Therefore, 1.04–1.18% of the raw reads were removed from our sequencing results. All the downstream analyses were based on the cleaned high quality sequence data. To map mRNA sequences to the mouse reference genome both, the reference genome (GRCm38/mm10) and the gene model annotation files were downloaded from the genome website. An index of the reference genome was built using Hisat2 v2.0.5 software and paired-end clean reads were aligned to the reference genome. The mapped reads of each sample were assembled by StringTie (v1.3.3b) (Mihaela Pertea.et al.2015) in a reference-based approach. For quantification of gene expression level features Counts v1.5.0-p3 sofware was used to count the read numbers mapped to each gene. Expected number of Fragments Per Kilobase of transcript sequence per millions base pairs sequenced (FPKM) of each gene was calculated based on the length of the gene and reads count mapped to this gene. Differential expression analysis of the two groups [GRSF1KO (n = 3) and WT (n = 4)] was performed using the DESeq2 R package (1.20.0). Both the unadjusted and the Benjamini and Hochberg (BH) adjusted p-values were reported.

### Proteome analysis including LC–MS/MS

Testicular tissue was homogenized in 8 M urea, 50 mM ammonium bicarbonate, supplemented with 1 × Halt protease inhibitor cocktail (Thermofisher Scientific, Henningsdorf, Germany). Samples were first homogenized at 30 Hz for 1 min, then sonicated, reduced with 5 mM Dithiothreitol (DTT, Thermo Fisher Scientific, Henningsdorf, Germany) and alkylated using 10 mM Iodoacetamide (IAA, Sigma Aldrich, St. Louis, USA). Overnight digestion was carried out using MS approved trypsin (Serva, Heidelberg, Germany) at 1:50 enzyme to substrate ratio at 37 °C. Tryptic peptides were acidified using formic acid to adjust to pH 2–3. The acidified tryptic digests were fractionated and desalted using high pH reverse phase fractionation in stage tips. Then 1 µg of total protein was used for LC–MS/MS analysis. Liquid-chromatography-coupled to tandem mass spectrometry (LC–MS/MS) was carried out on a quadrupole-ion-trap-orbitrap MS (Orbitrap Fusion, Thermo Fisher) operated in positive ion mode coupled to a nano-UPLC (Dionex Ultimate 3000 UPLC system, Thermofisher, Henningsdorf, Germany). Peptides were separated using a 140-min gradient with linearly increasing ACN concentration from 2 to 30% ACN in 120 min. Raw files were searched, quantified and normalized using the MaxQuant software package version 1.6.17.0 [27] with default settings for orbitraps. The match between runs (MBR) feature was disabled but the LFQ (Label-Free Quantification), iBAQ (Intensity Based Absolute Quantification) and classical normalization features were enabled. We used the UniProt mouse reference proteome as database (downloaded in November 2021 with 21994 entries) with enzyme specificity set to Trypsin/P, cysteine carbamidomethylation as a fixed modification (+ 57.021464) and methionine oxidation (+ 15.994914) as well as protein N-terminal acetylation (+ 42.010565) were as variable modifications. Data analysis was performed using the R 4.1.2 and Rstudio 1.4.1106 programs [28, 29].

### Statistic gene overrepresentation analysis

Based on the unadjusted p < 0.05 Gene overrepresentation analysis (ORA) was carried out using clusterProfiler V4.2.2 within R 4.1.2 and Rstudio 1.4.1106 programs [30]. For RNAseq, all 563 differentially expressed genes were used as input gene list **(**Additional file 1: Table S1) and the gene background/universe consisted of 33663 genes from RNAseq analysis (Additional file 2: Table S2). GO-term analysis was carried out using GO-CC, GO-MF, and GO-BP with organism specificity set to ‘mus musculus’, the keyType set to ‘ENSEMBL’, pvalueCutoff off set to 1. To reduce GO-term redundancy, terms with at least 0.7 semantic similarity were combined via ClusterProfiler:simplify and the top five terms by GeneRatio were plotted as a bubbleplot for each GO category (CC, BP, MF). Enrichment analysis for proteomic data were carried out using an analogous approach. 302 differentially expressed proteins (unadjusted p-value of a t-test) were used as input gene list. The background universe consisted of 5167 proteins detected using mass spectrometric analysis. ClusterProfiler parameters were identical between proteomic and RNAseq ORA analysis, apart from keyType set to ‘SYMBOL’.

### Creation of Grsf1 deficient mice

For preparation of conditional *Grsf1* knockout mice a knockout construct was created by the European Conditional Mouse Mutagenesis (EUCOMM) program (Fig. 1). In this construct exons 4 and 5, which encode for RNA binding domain qRRM1, were flanked by recognition sequences for Cre-recombinase (*loxP* sites). Furthermore, the construct involved an artificial exon between exons E3 and E4. This exon involved a functional LacZ construct and a neomycin resistance minigene. The LacZ construct started with a splice acceptor site (En2 SA) and involved downstream an internal ribosomal insertion site (IRES), an open reading frame for the ß-galactosidase and a polyadenylation signal. Immediately downstream of this polyadenylation sequence a further *loxP* sequence and a neomycin resistance minigene was included. This minigene involved of a human beta-actin promoter, a neomycin resistance cassette and a polyadenylation signal. The LacZ construct and the neomycin resistance minigene are flanked by two flippase recognition target (FRT) recombinase recognition sites. Successful integration of this gene construct into the genome of target cells allows survival of the cells in neomycin containing media. FRT recombination deletes the lacZ gene and the neomycin minigene from the genome of selected cells and establishes the conditionality of the construct. The selected cells carry a functional Grsf1 gene although the *loxP* sequences are still present upstream of exon E4 and downstream exon E5. From the functional point of view this genotype represents wildtype mice, which carry a fully functional *Grsf1* gene although exons E4 and E5 are still flanked by the *loxP* recognition sequences. When these animals were crossed with mice expressing the cre-recombinase under the control of the CMV-promoter exons E4 and 5 are deleted from the genome in all cells. This genomic deletion leads to the formation of a premature stop codon, when the mutant *Grsf1* gene is transcribed and the truncated *Grsf1* mRNA is degraded via nonsense-mediated decay [31]**.**

Employing this strategy, we created heterozygous *Grsf1*-deficient mice (*Grsf1*^±^), which carry one mutant (*Grsf1*^*−*^ allele) and one wildtype allele (*Grsf1*^+^*)* allele. These heterozygous allele carrier (*Grsf1*^±^) were subsequently mated and homozygous *Grsf1*^−/−^ mice were selected. These founder animals were crossbred and a colony of *Grsf1*^−/−^ mice was established by intercrossing homozygous *Grsf1*^*−/−*^ mice. For characterization experiments these homozygous *Grsf1*^−/−^ mice were compared with commercial C57Bl/6J mice.

### Tissue lysate preparation and immunoblotting

Homozygous *Grsf1*^*−/−*^ mice and wildtype control animals were sacrificed under halotane anesthesia by cervical dislocation. The major organs were prepared and 50 mg wet weight of the tissues were homogenized in 0.5 ml PBS using a MP FastPrep24 tissue homogenized (MB Biomedicals, Irvine, USA). The tissue homogenate was centrifuged for 10 min at 20,000*g* and the tissue lysate supernatants were analysed for the presence of GRSF1 protein by immunoblotting. For this purpose aliquots (3–5 µl) of the tissue lysate supernatants involving 40 µg protein were applied to SDS-PAGE on a 7.5% SDS-PAGE analytical gel that was overlaid with a 4% SDS-PAGE stacking gel. After electrophoresis the separated proteins were transferred to a nitrocellulose membrane (Serva Electrophoresis GmbH, Heidelberg, Germany), which was blocked overnight with the BlueBlock solution (Serva Electrophoresis GmbH, Heidelberg, Germany). After washing with PBS the blot was first incubated for 1 h at room temparature with anti-GRSF1 antibodies. For probing the blot of testis lysate supernatants the rabbit anti-GRSF1 antibody AB 205531 (Abcam, Cambrifge, UK) was used as primary antibody at a 1:1000 dilution. For other organ lysate supernatants, the rabbit anti-GRSF1 antibody HPA036985 (Sigma, Deisenhofen, Germany) was employed at a 1:1000 dilution. After incubation with the rabbit primary anti-GRSF1 antibodies the blots were rinsed with PBS and were subseqeuently incubated overnight with a POD-containing anti-rabbit IgG antibody (Sigma, Deisenhofen, Germany, MFCC00162788, raised in goat, 1:4000 dilution in BlusBlock solution) at 4 ℃. The membrane was washed twice with PBS and the blot was finally developed with the SERVA*Light* Polaris CL HRP WB substrate kit for 5 min at room temperature.

After digitalization the membrane was stripped using the BlueClear solution (Serva Electrophoresis GmbH, Heidelberg, Germany). For this purpose, the BlueClear solution was warmed up to 55° C and incubated with the membrane for 30 min under gentle agitation. Then the membrane was washed three times with PBS containing 0.3% Tween 20 and was blocked again with BlueBlock solution (Serva Electrophoresis GmbH, Heidelberg, Germany) for 30 min at room temparature. Next, a anti-GAPDH antibody MA5-15738 (Invitrogen, Waltham, USA) was added at 1:2000 dilution in BlueBlock solution and the membrane was incubated in this solution for 1 h at room temparature. After washing with PBS the mebrane was incubated for 1 h at room temperature with a POD-labeled anti-mouse IgG (Sigma, Deisenhofen, Germany, 1:5000 dilution in BlueBlock solution). Then the membrane was washed twice with PBS and the blot was finally developed with the SERVA*Light* Polaris CL HRP WB substrate kit (Serva Electrophoresis GmbH, Heidelberg, Germany) for 5 min at room temperature.

### In vivo aging and body weight kinetics

Male and female *Grsf1*^−/−^ mice and wildtype control animals (C57BL/6 mice, n = 10 in each experimental group) were housed in separate cages with water and standard chow diet ad libitum. The body weights were taken once a week over the time period indicated. The body weight kinetics were visualized using the GraphPad Prism version 8.2.0 for Windows (GraphPad Software, San Diego, USA,) and statistic evaluation was carried out using the Wilcoxon test.

### Blood withdrawal and determination of basic hematological parameters

For quantification of the basic hematological parameters, 18–20 months old male and females *Grsf1*^−/−^ mice and age- and gender-match wildtype control animals (C57BL/6 mice) were used. Animal were sacrificed under anesthesia by cervical dislocation and EDTA blood was obtained by heart puncture. The basic hematological parameters (Hb, HK, erythrocyte count, leucocyte count, MCV, MHC, MCHC) of the two genotypes in three different age groups were determined at the Institut für Veterinärmedizinische Diagnostik GmbH (Berlin, Germany).

### Preparation of bone marrow cells

For quantifying the expression of *Grsf1* mRNA by qRT-PCR we also prepared bone marrow cells of *Grsf1*^−/−^ mice and wildtype control animals (C57BL/6 mice). For this purpose, mice of either genotype were sacrificed under isoflurane anesthesia by cervical dislocation and the two femur bones were prepared from each individual. Adhering connective and muscle tissue was carefully removed and the two ends of the bones were cut-off. The bone marrow cavity was punctured and bone marrow cells were washed out with 10 ml of PBS for each bone. The cell suspensions were combined, the cells were pelleted at 500 × g and were washed twice with PBS. Finally, the cells were reconstituted in 0.6 ml of PBS and total RNA was extracted.

## Results

### Creation of Grsf1-deficient mice

The mouse *Grsf1* gene spans about 17 kbp, consists of 10 exons and 9 introns and has been mapped to a central region of chromosome 5. The functionally relevant RNA binding domains (qRRMs) are encoded for by exons 2–9. For conditional knockout of the *Grsf1* gene a floxed mutant construct (Fig. 1) was created by the European Conditional Mouse Mutagenesis (EUCOMM) program. For preparation of this construct an artificial exon was cloned between exons 3 and 4 of the *Grsf1* gene. Mouse embryonic stem cells were transfected with this construct. Cells that have successfully incorporated the construct into the genome via homologous recombination were selected and used for blastocyst injection. The resulting chimera were screened for germline transmission of the knockout construct and homozygous *Grsf1*-floxed mice were bred. These mice were mated with FLP-deleter mice yielding individuals, in which the sequences located between the two FRT sites was removed. These individuals carry quasi-wildtype *Grsf1* alleles with an open reading frame for the entire protein. The only genotypic difference between individuals carrying the mutant *Grsf1* alleles and those carrying the true wildtype *Grsf1* alleles was that in the mutant mice exons 4 and 5 were flanked by two unidirectional *loxP* sites. These additional sequences were localized in introns 3 and 5 and thus, were not of functional relevance for protein expression. When these mice were crossed with Cre-deleter mice, in which the expression of the Cre-recombinase is governed by the CMV promoter [B6.C-Tg(CMV-cre)1Cgn/J], the sequences localized between the two *loxP* sites were removed in all cells. In these mice the *Grsf1* gene is truncated (lacking exons E4 and E5) and its expression yields incomplete transcripts that rapidly undergo nonsense mediated decay [31].

For more detailed functional characterization, we bred a colony of *Grsf1*^−/−^ mice and did not observe major phenotypic alterations. The mice were fertile, the embryos developed normally, and we did not observe any peculiarities during delivery. Offspring developed normally and we did not see major differences between wildtype mice and *Grsf1*^−/−^ individuals during the early post-natal period.

### *Grsf1* mRNA is present at high copy numbers in testis, liver and kidney of wildtype mice but absent in these organs of *Grsf1*^*−/−*^ individuals

**Fig. 2:**
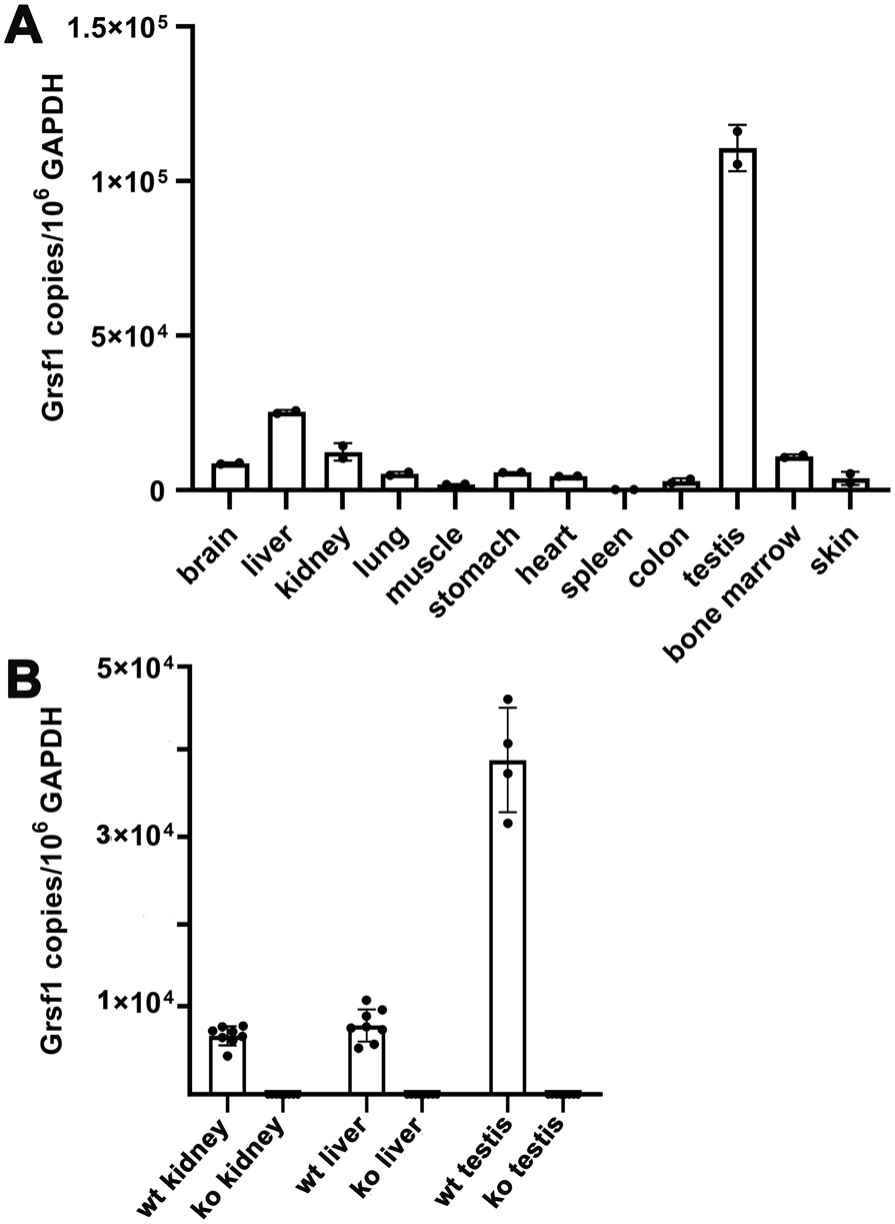
Expression of *Grsf1* mRNA in different tissues of wildtype mice and *Grsf1*^−/−^ animals. Total RNA was extracted from different tissues of wildtype mice (C57Bl/6J) and *Grsf1*^−/−^ animals. *Grsf1* mRNA was quantified by qRT-PCR using a specific primer combination as described in Materials and Methods. The copy numbers of *Grsf1* mRNA per 10^6^ Gapdh mRNA copies were calculated and used as measure for the steady-state concentrations of* Grsf1* mRNA. **A** Expression of *Grsf1* mRNA in different cells and tissues of C57Bl/6J wildtype mice, **B** comparative analysis of the *Grsf1* mRNA concentrations in selected organs of C57Bl/6J wildtype mice (wt) and grsf1^−/−^ individuals (ko)

To confirm that our *Grsf1*^−/−^ mice do not express the truncated *Grsf1* gene we first profiled *Grsf1* mRNA expression in different organs of wildtype mice using qRT-PCR. Since our method involves amplification of externally added amplification standards (amplicons) we were able to quantify the copy numbers of *Grsf1* mRNA and *Gapdh* mRNA in a given volume of our RNA preparation. Here we found that *Grsf1* mRNA is present in almost all tested tissues at low steady state concentrations (Fig. 2A). Although we also detected *Grsf1* mRNA copies in skeletal muscle the copy numbers in this tissue were not particularly high. This finding was somewhat surprising since earlier expression profiles suggested higher *Grsf1* expression levels in skeletal muscles [25]. It should, however, be stressed that in that study the relative *Grsf1* mRNA concentrations were quantified during in vitro myoblast differentiations and thus, no clear-cut conclusions can be drawn from these experimental data on the absolute expression levels of *Grsf1* mRNA in the skeletal muscles of wildtype mice. Using our more quantitative qRT-PCR approach we detected higher steady-state concentrations of *Grsf1* mRNA in testis, liver and kidney but also in brain and bone marrow cells (Fig. 2A).

In *Grsf1*^−/−^ mice no *Grsf1* mRNA should be present and we confirmed this experimentally for kidney, liver and testis (Fig. 2B). In fact, even at high cycle numbers (40 amplification cycles) no specific *Grsf1* signals could be detected in these organs. These data indicate that our knockout strategy abolished *Grsf1* mRNA expression in the *Grsf1*^−/−^ mice.

### GRSF1 protein expression is abolished in different organs of *Grsf1*^*−/−*^ mice

After showing that the functional inactivation of the *Grsf1* gene led to an abolished Grsf1 mRNA expression (Fig. 2B) we quantified the steady-state concentrations of the GRSF1 protein. For this purpose we analyzed homogenate supernatants of different organs with two anti-GRSF1 antibodies.

**Fig. 3:**
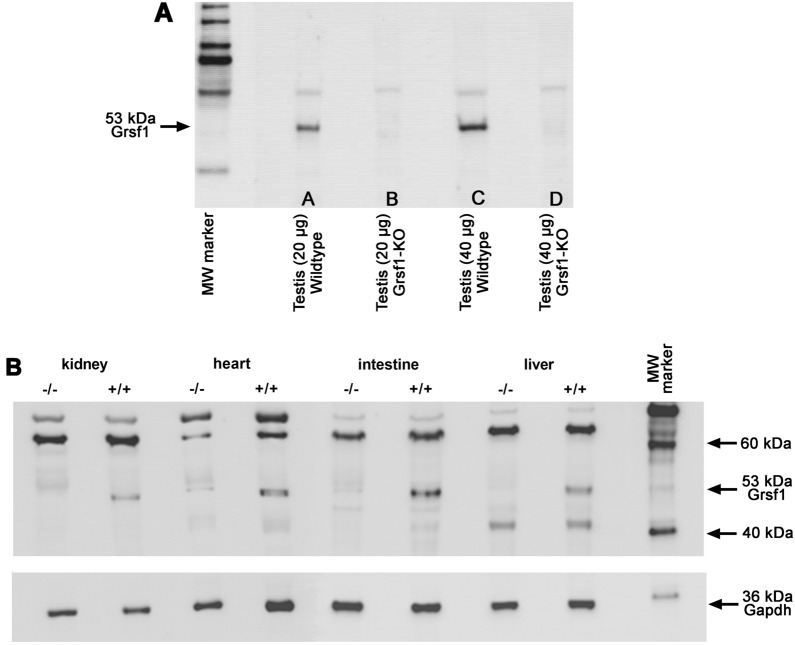
Expression of GRSF1 protein in different tissues of wildtype mice and *Grsf1*^−/−^ animals. Different organs were prepared from sacrificed mice of either genotype. Small pieces of the different organs were homogenized. The homogenates were centrifuged and aliquots of the supernatants were applied to SDS-PAGE. Immunostaining was carried out with two different anti-GRSF1 antibodies. **A** Testis homogenate supernatants: Here 45 µg total testis lysate supernatant protein were applied and the anti-GRSF1 antibody AB 205531 (Abcam, Cambrifge, UK) was used at primary antibody. **B** Organ homogenate supernatants: Here 40 µg total homogenate supernatant protein were applied and the anti-GRSF1 antibody HPA036985 (Sigma, Deisenhofen, Germany) was used as primary antibody. For comparative purpose Gapdh was immunostained on the same blots after stripping the membrane (lower panel)

As shown in Fig. 3A a clearly visible immunoreactive band that migrated at 53 kDa (predicted MW of GRSF1) was detected when testis homogenate supernatants were analyzed (lane A). When we doubled the protein amount a more intense signal was detected (lane C). In contrast, when testis homogenates of *Grsf1*^−/−^ mice were analyzed no immunoreactive bands were observed in this molecular weight region independent of the amounts of protein loaded on SDS-PAGE (lanes B + D).

Since GRSF1 is not only expressed in testis but also in other cells and tissues (Fig. 2A) we attempted to detect the GRSF1 protein also in the homogenate supernatants of kidney, heart, small intestine and liver. Using the same antibody as in the testis analysis (Fig. 3A) we detected a large number of immunoreactive bands. Thus, we decided to use another anti-GRSF1 antibody (HPA036985 from Sigma, Deisenhofen, Germany). With this antibody we observed a much less complex pattern of immunoreactive bands, which included a band in the molecular weight range of GRSF1 (53 kDa). When we used homogenate supernatants prepared from the organs of *Grsf1*^*−/−*^ mice this band was not visible indicating functional inactivation of the *Grsf1* gene in our *Grsf1*^−/−^ mice. For control purpose we quantified GAPDH protein expression using the same blots after stripping of the nitrocellulose blotting membrane (Fig. 3B, lower panel). Here we detected similar GAPDH concentrations in the homogenate supernatants of wildtype and *Grsf1*^*−/−*^ organs.

### Aging male *Grsf1*^*−/−*^ mice gain significantly less body weight than wildtype controls

**Fig. 4:**
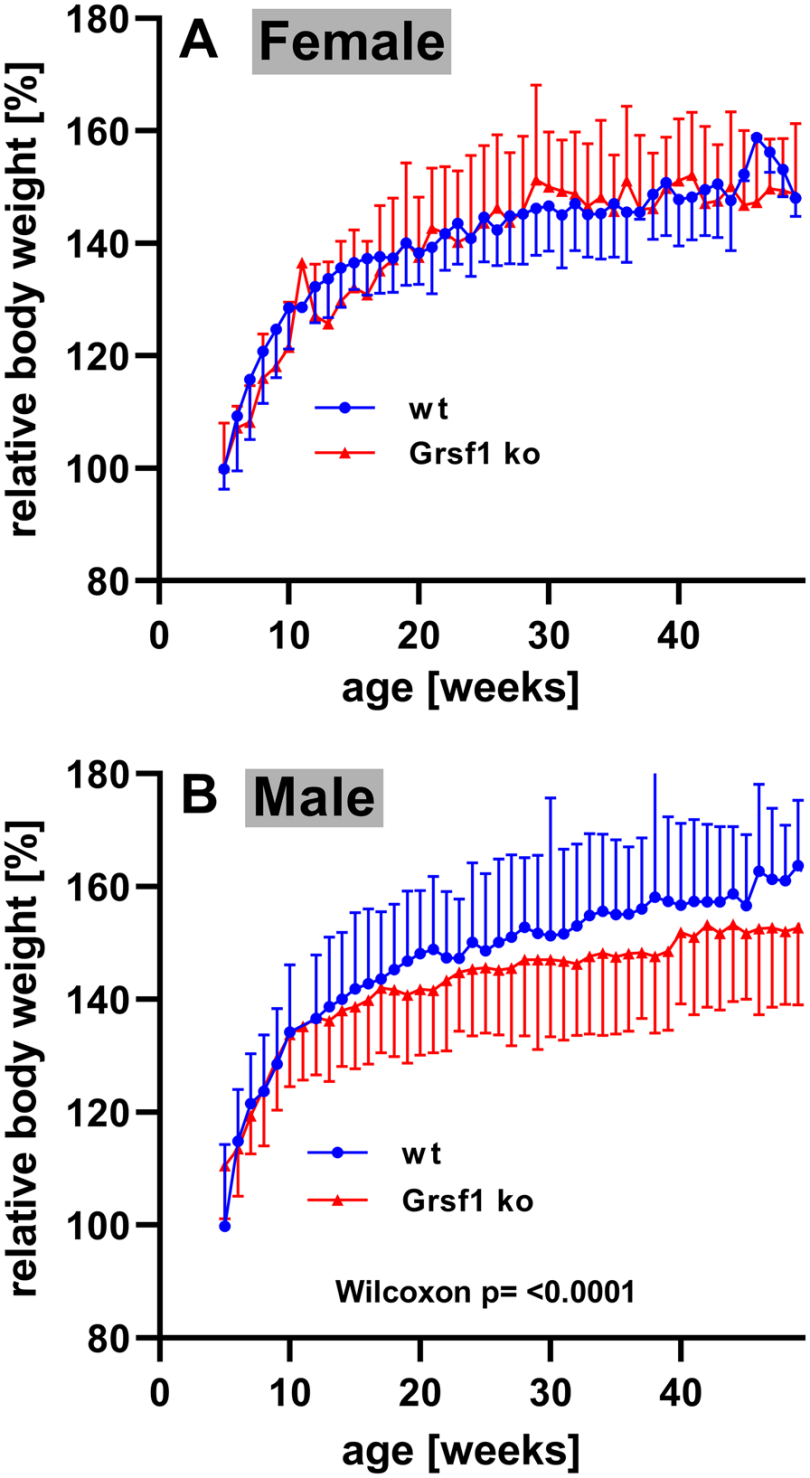
Body weight kinetics of *Grsf1*^−/−^ mice and corresponding C57Bl/6J wildtype controls. From our colony of *Grsf1*^−/−^ mice and from wildtype control animals (C57Bl/6J) 10 male and 10 female individuals with similar body weights were selected. Of each genotype the males and the females were housed in separate cages. The animals were allowed to age for up to 50 weeks with standard chow diet and water *at libitum*. Each week the individual body weights were measured once and the animals were inspected for phenotypic peculiarities. The body weight of each individual at the beginning of the experiment was set 100% and the relative bodyweight increase was quantified. **A** Female individuals, **B** Male individuals

As described above *Grsf1*^−/−^ mice were viable and young individuals did not show major developmental defects. To explore whether such defects will show up in later developmental periods we followed the body weight kinetics of *Grsf1*^*−/−*^ mice and C57Bl/6J wildtype controls over a time period of up to 50 weeks (Fig. 3). Here we found that the body weight kinetics of female *Grsf1*^−/−^ mice were almost identical with those of wildtype controls. In fact, the two growth curves were almost superimposable (Fig. 4 A). When we followed the body weight kinetics of male individuals, we also observed identical growth curves for *Grsf1*^*−/−*^ mice and wildtype controls during the first 15 weeks (Fig. 4B) and statistic evaluation (Wilcoxon test) of the experimental raw data indicated no significant difference between the two genotypes in this time window. However, at later time points *Grsf1*^*−/−*^ mice gained significantly less body weight than the corresponding control animals. Although these differences were not dramatic (the means of the knockout mice are about 10% lower than those of the control animals in the time window > 40 weeks) they were statistically highly significant (Wilcoxon-test, p < 0.001). It should be stressed that all (n = 10 in each group) individuals in the four experimental groups (female *Grsf1*^*−/−*^, female C57Bl/6J wildtype, male *Grsf1*^*−/−*^ and C57Bl/6J wildtype) survived the aging period in good health. In fact, we did not observe obvious differences between the two genotypes when overall mobility, social behavior and fur appearance were evaluated as readout parameters.

### *Grsf1* deficiency does not compromize the hematopoietic system

**Fig. 5:**
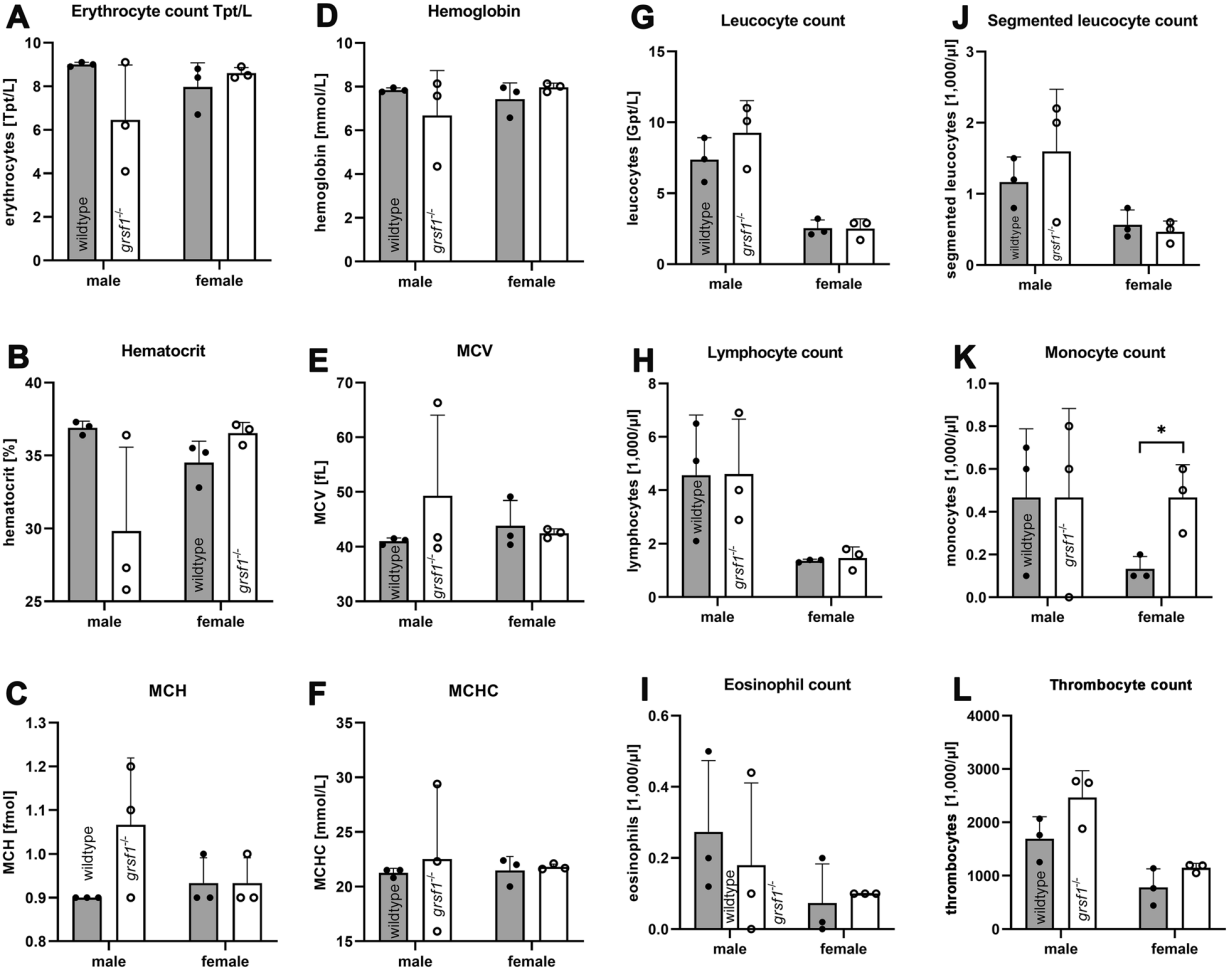
Comparison of basic hematological parameters of *Grsf1*^*−/−*^ mice and corresponding C57Bl/6J wildtype controls. The experiment was carried out as described in the Material and Method section. Mice were sacrificed, blood was removed by cardiac puncture and basic hematological parameters were determined

Previous in vitro experiments have implicated GRSF1 in red blood cell development [19]. If this effect may be of in vivo relevance and if there are no sufficient compensation reactions we reasoned that our *Grsf1*^*−/−*^ mice would suffer from anemia. To test this hypothesis we sacrificed three individuals of each experimental group at the end of the aging period and recovered EDTA blood by heart puncture. Basic hematological parameters [erythrocyte count (Ery), hematocrit (HK), mean corpuscular hemoglobin content (MCH), hemoglobin concentration (Hb), mean corpuscular volume (MCV), mean corpuscular hemoglobin concentration (MCHC), lymphocyte count, eosinophil count, segmented granulocyte count, monocyte count (Mono), thrombocyte count were determined. Most of these basic hematological parameters were not significantly different between *Grsf1*^*−/−*^ mice and wildtype controls (Fig. 5). The monocyte count of femal individuals (Fig. 5K) was the only exception. Here we found that *Grsf1*^*−/−*^ mice had significantly (t-test, p = 0.049) elevated monocyte counts when compared with wildtype female controls. Despite this difference the determined hematological data suggest that systemic functional inactivation of the *Grsf1* gene does not compromize the hematopoietic system. Thus, Grsf1 may not play a major role in the hematopoiesis of adult mice or the potential defect is well compensated under in vivo conditions.

### Testicular transcriptome analysis

GRSF1 is an RNA binding protein that has been implicated in post-transcriptional processing of primary RNA transcripts as well as in mRNA transport [32–35]. Thus, systemic functional inactivation of the *Grsf1* gene may modify cellular transcriptomes. Since we observed high level expression of *Grsf1* in testis we compared the testicular transcriptomes of *Grsf1*^*−/−*^ mice and C57Bl/6J wildtype controls. For this purpose, we extracted total RNA from 3 different *Grsf1*^*−/−*^ mice and 4 wildtype controls and analyzed the differences in the steady state concentrations of some 33,000 transcripts (Additional file 2: Table S2). Based on the BH-adjusted p-value of < 0.05, only *Grsf1* mRNA was found to be differentially expressed. In wildtype control testis this mRNA is present in well detectable concentrations, whereas in *Grsf1*^*−/−*^ testis this mRNA was hardly detectable. These data confirmed our qRT-PCR results (Fig. 2) indicating that functional inactivation of the *Grsf1* gene completely abolishes its expression.

**Fig. 6:**
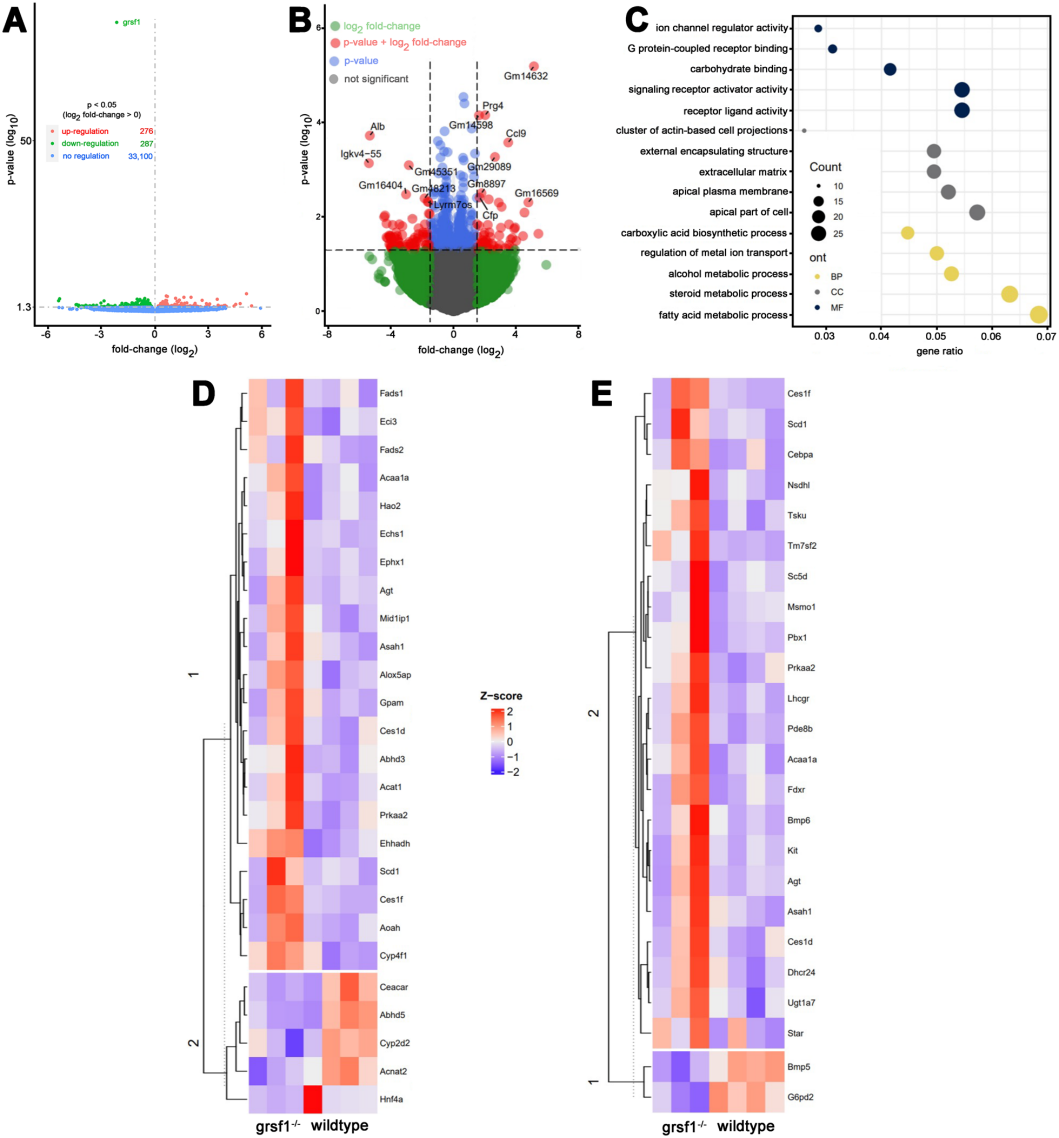
Comparison of the testicular transcriptomes of *Grsf1*^*−/−*^ mice and wildtype controls. Total RNA was extracted from testes of G*rsf1*^*−/−*^ mice (n = 3) and wildtype controls [C57Bl/6, n = 4)]. The transcriptomes were quantified as described in the Materials and Methods section. **A** 287 transcripts including *Grsf1* were downregulated (blue), 276 transcripts were upregulated in *Grsf1*^*−/−*^ mice (red) whereas 33,100 transcripts were not significantly different between the two genotypes (blue). **B** Same as **A**, with y-axis truncated at − log10(10e−7). Transcripts with an absolute log2 fold-change of > 1.5 and an unadjusted p < 0.05 are shown in red. Transcripts with an absolute log2 fold-change < 1.5and an unadjusted p < 0.05 are depicted in blue. **C** Overrepresentation analysis was performed as described in the Materials and Methods sections and gene ontology categories are shown. All 563 differentially expressed genes (based on unadjusted p-value of < 0.05) were used as input gene list (Additional file 1: Table S1) and the gene background/universe consisted of 33,663 genes from RNAseq analysis (Additional file 2: Table S2). The top five GO terms per category based on their gene ratio (k/n with k = genes of this cluster belonging to GO term and n = genes of all clusters belonging to GO term) are shown. Biological processes (BP) are depicted in yellow, cellular component (CC) in grey and molecular function in dark blue. **D** Hierarchically clustered heatmaps of regulated transcripts (unadjusted p < 0.05) of steroid metabolic process. **E** Hierarchically clustered heatmaps of regulated transcripts (unadjusted p < 0.05) of fatty acid metabolic process. Heatmap colours indicate z-score scaled gene expression values, with blue for low and red for high expression levels

Since we observed biological heterogeneity in the testicular transcriptome of *Grsf1* knockout mice (Fig. 6D/E), further analyses were of exploratory nature only and carried out using unadjusted p-value cut-offs. We acknowledge that due to the use of unadjusted p-values, limited biological insights can be gleaned from these analyses. The tissue concentrations of the vast majority of testicular transcripts (> 98%) were not different when *Grsf1*^*−/−*^ mice and wildtype controls (Fig. 6A, depicted in grey and green) were compared. In Fig. 6B differential testicular gene expression of *Grsf1*^*−/−*^ mice and wildtype C57Bl/6J control animals is visualized in more detail.

In black, those transcripts are shown, for which no differential expression was observed when *Grsf1*^*−/−*^ testis was compared with the same organ of wildtype control animals (unadjusted p > 0.05). In blue, those transcripts are shown that were differentially regulated (unadjusted p < 0.05) but the fold change of which was less than 1.5 (429 transcripts). In red, differentially regulated transcripts (unadjusted p < 0.05) with a fold change of more than + 1.5 (54 transcripts, upregulation) or less than − 1.5 (79 transcripts), downregulation) are shown. Next, we performed over-representation analysis of the regulated transcripts (unadjusted p < 0.05) and found the gene ontology classes “fatty acid metabolic processes” and “steroid metabolic process” overrepresented (Fig. 6C). A more detailed view of the differentially regulated transcripts is given in Fig. 6D (fatty acid metabolic processes) and Fig. 6E (steroid metabolic process). While on average, the groups seem to separate by these transcripts (unadjusted p < 0.05), one of the three *Grsf1* knockout mice generally expressed lower amounts of transcript related to i) steroid metabolic process and ii) fatty acid metabolic process. We also observed some of the biological variation in the *Grsf1* wildtype mice as well, particularly in the steroid metabolic process.

Next, we compared our transcriptome dataset with an existing microarray sequencing dataset generated from RNA extracts from two different skeleton muscles (*quadriceps femoris*, *rectus femoris*) of wildtype and muscle-specific *Grsf1*^−/−^ mice [25]. This comparison only involves the significantly regulated transcripts detected in the two studies. Of the 249 differentially regulated mRNAs observed in the previous study [25] we only detected four transcripts in our transcriptome analysis highlighting the tissue specific gene expression profiles between testis and muscle. Comparing our differentially expressed genes with those in the previous dataset [25], we found *Grsf1* and *Ptger3*, *Slc14a2*, *Rab15* to be constitently regulated.

### Testicular proteome analysis

Previous studies suggested that the GRSF1 protein binds to different RNAs regulating expression of these transcripts [19–22]. For instance, GRSF1 has been implicated in translational regulation of *Gpx4* expression, which is expressed in high levels in spermatoid cells [36–38]. Moreover, GRSF1 is required for translation of the mRNA encoding for the *SNARE protein Use1* [19]. If the regulatory activity of GRSF1 is essential for testicular GPX4 expression, the GPX4 protein should be present in testicular protein extracts at much lower concentrations when *Grsf1*^*−/−*^ mice are compared with wildtype control animals.

**Fig. 7:**
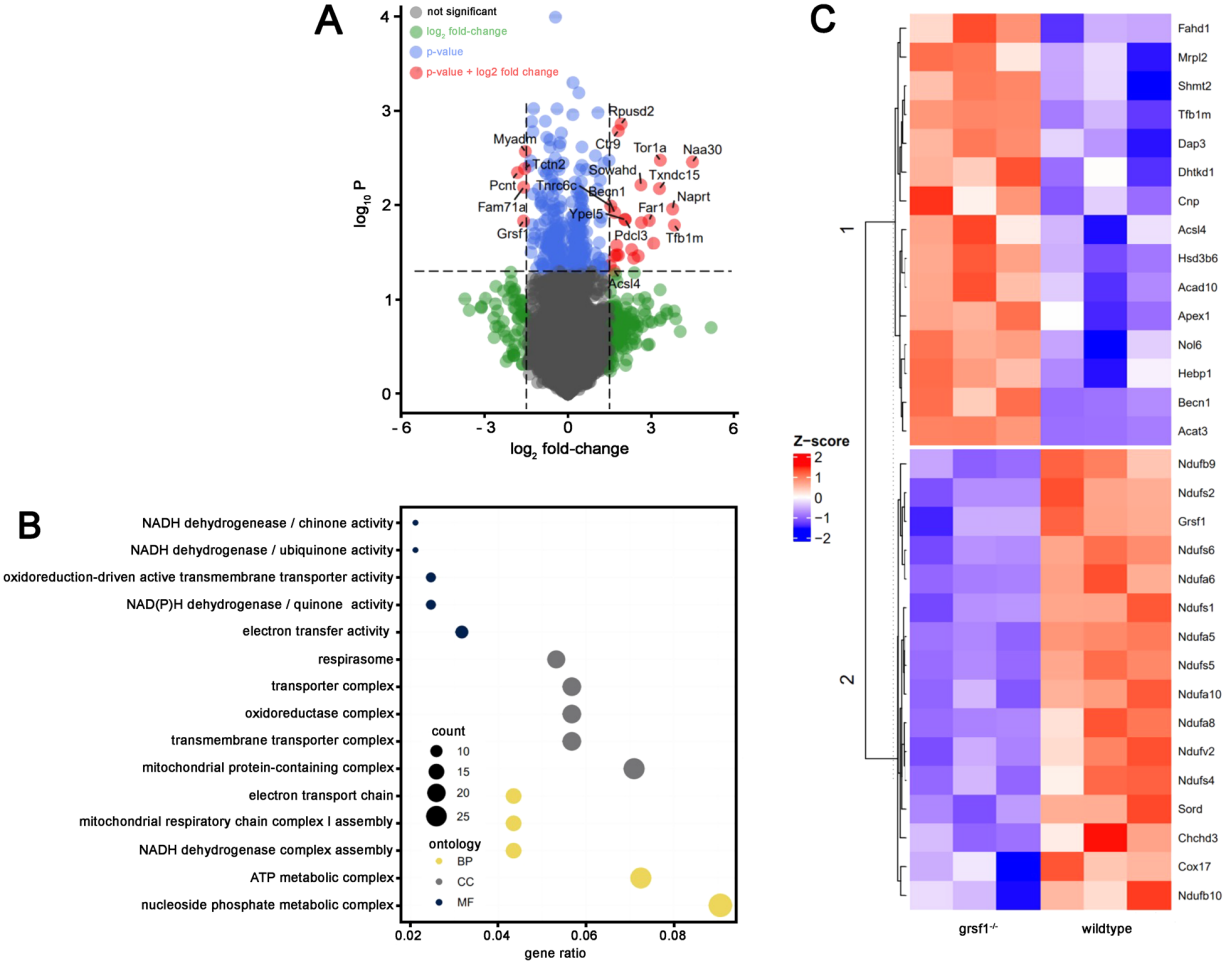
Comparison of the testicular proteome of *Grsf1*^*−/−*^ mice and wildtype controls. Total proteins were extracted from testes of three G*rsf1*^*−/−*^ mice and three wildtype controls and the proteomes were quantified as described in the Materials and Methods section. **A** 5167 proteins were detected using mass spectrometry. Of these, 302 proteins were differentially regulated based on an unadjusted t-test p < 0.05 (blue and red). In red are those proteins with an absolute log2 fold-change > 1.5. Proteins below the p-value cut-off but with an absolute log2 fold-change > 1.5are shown in green. **B** Overrepresentation analysis of GO-terms. 302 differentially expressed proteins (unadjusted p-value) were used as input protein list. The background universe consisted of 5167 proteins detected using mass spectrometric analysis. The top five GO terms per category based on their gene ratio (k/n with k = genes of this cluster belonging to GO term and n = genes of all clusters belonging to GO term) are shown. Biological processes (BP) are depicted in yellow, cellular component (CC) in grey and molecular function in dark blue. **C** Hierarchically clustered heatmaps of regulated proteins (unadjusted p < 0.05 and log2 fold-change > 0.5) of associated with the GO term ‘mitochondrion’ (GO:0005739). Colours indicate z-score scaled protein expression values, with blue for low and red for high expression levels

To assess the effect of *Grsf1* knock out on GPX4 protein concentrations, we carried out deep proteomic analysis of testicular protein extracts prepared from *Grsf1*^*−/−*^ mice and wildtype controls (C57BL/6). We quantified the steady-state concentrations of more than 5,100 different testicular proteins (full dataset available at https://www.ebi.ac.uk/pride PXD034916). GRSF1 was detected in wildtype controls, but not in knockout mice. No other protein was significantly altered. Using unadjusted p-values, 158 proteins (3.0%) were upregulated (unadjusted p < 0.05) and 144 (2.8%) were downregulated (unadjusted p < 0.05) (Fig. 7A). Since GRSF1 has been implicated in translational regulation of *Gpx4* and *Use1* mRNA we specifically searched our testicular proteomes for tryptic digest fragments of GPX4 and USE1. GPX4 is highly expressed in spermatoid cells [36, 39] and we detected GPX4 specific digest fragments in the testicular protein extracts of both Grsf1^*−/−*^ mice and wildtype control animals. However, we did not detect differences in the steady-state GPX4 concentrations between the two genotypes. Thus, systemic functional inactivation of the *Grsf1* gene does not dramatically impact testicular GPX4 expression. The SNARE protein USE1 was not among the quantifyable proteins in the testicular protein extracts, precluding assessment of whether systemic functional inactivation of the *Grsf1* gene in vivo might alter the expression of this protein. For this purpose protein extracts obtained from other organs should be explored.

To investigate the biological relevance of the observed proteomic differences between *Grsf1*^*−/−*^ mice and wildtype controls we carried out gene ontology enrichment analysis. Among the most regulated biological processes were the nucleoside phosphate metabolic process, the ATP metabolic process and the mitochondrial protein-containing complex. Interestingly, many of these gene ontology categories were related to mitochondrial processes, such as NADH dehydrogenase-quinone activity, NADH dehydrogenase-ubiquinone activity, oxidoreduction-driven active transmembrane transporter acticity, NAD(P)H dehydrogenase-quinone activity, electron transfer activity, respirasome, oxidoreductase complex, mitocondrial protein-containing complex, electron transport chain, mitochondrial respiratory chain complex I assembly, NADH dehydrogenase complex assemby and ATP metabolic process (Fig. 7B). This data consistent with previous reports about the involvement of GRSF1 in mitochondrial functionality [11, 12, 26]. To obtain more detailed information about the functional alterations induced by systemic inactivation of the Grsf1 gene we performed hierarchical clustering of mitochondrial proteins that were differentially expressed in our analysis (Fig. 7C). Interestingly, many of the significantly downregulated proteins (NDUFB9, NDUFS2, NDUFS6, NDUFA6, NDUFS1, NDUFA5, NDUFS5, NDUFA10, NDUFA8, NDUFV2, NDUFS4, NDUFB10) are constituents of complex I of the mitochondrial respiratory chain. Although we did not test the functionality of the respiratory chain, based on our proteome data, electron transfer through complex I might be compromised.

## Discussion

### Degree of novelty and limitations of the present study

GRSF1 was first described in 1994 as poly(A) + mRNA binding protein which interacts with a conserved G-rich sequence element in RNAs [16]. To date, there are over 50 published studies on GRSF1 implicating this RNA binding protein in a number of physiological processes including RNA processing, redox homeostasis, embryo development, brain development erythropoiesis, stem cell differentiation, carcinogenesis and in viral infections [8]. However, due to the absence of any knockout mouse models, the physiological function of GRSF1 remains poorly understood. Only recently, Driscoll et al*.* reported the creation of muscle-specific *Grsf1*^*−/−*^ mice (conditional knockout mice) [25]. To obtain these animals floxed *Grsf1* mice, in which exons 4 and 5 of the *Grsf1* gene were framed by *loxP* recognition sequences, were crossed with *Myf5-Cre* mice, which specifically deleted exons 4 and 5 from the *Grsf1* gene in all skeletal muscles. These mice were then tested for muscle endurance but no difference was observed between *Grsf1*^*−/−*^ mice and wildtype controls when 2–4 months old or 7–9 months old individuals were compared. In contrast, 16–18 months old *Grsf1*^*−/−*^ mice displayed significantly reduced muscle endurance. Despite this functional difference, histologically, both genotypes showed normal muscle morphology with similar age-appropriate signs of structural degeneration [25].

For the present study we followed a similar research strategy but crossed our floxed *Grsf1* mice with Cre-deleter mice, in which the expression of the Cre-recombinase is governed by the CMV promoter [B6.C-Tg(CMV-cre)1Cgn/J]. In the genomes of the resulting progeny the sequences localized between the two *loxP* sites were removed in all cells (systemic *Grsf1* knockout). In these mice the *Grsf1* gene is truncated and expression of the compromised gene yields incomplete transcripts that undergo nonsense mediated decay [31]. When we characterized our systemic *Grsf1*^*−/−*^ mice we found that these animals are viable, breed normally and do not show major signs of functional deficiency. However, with age male *Grsf1*^*−/−*^ individuals gained significantly less body weight than wildtype controls (Fig. 4B). Interestingly, this effect was gender-specific since we did not observe impaired weight gain in female *Grsf1*^*−/−*^ individuals (Fig. 4A).

One limitation of our study is the different genetic background of our *Grsf1*^*−/−*^ mice (mixed genetic background) and of the employed wildtype controls (C57Bl/6 genetic background). Thus, the relatively subtle growth retardations observed for male *Grsf1*^*−/−*^ mice (Fig. 4B) may in principle be related to differences in the genetic backgrounds of the two mouse lines. However, if background problems would be responsible for the observed weight differences between *Grsf1*^*−/−*^ and wildtype mice similar phenotypic alterations would be expected for female individuals. Since this was not the case (Fig. 4A) it is unlikely that the significantly different growth curves of aging male *Grsf1*^*−/−*^ mice are background related. The gender-specific differences in the body weight kinetics between male *Grsf1*^*−/−*^ mice and wildtype controls might be a consequence of an off-target effect of our genetic manipulation. Although introduction of the knockout construct was performed by homologous recombination, which is usually highly specific, modification of other parts of the genome cannot be ruled out completely. We did not carry out detailed off-target analysis and this is certainly a limitation of our study. Whole genome sequencing would help identify such off-target alterations but corresponding experiments have not been carried out.

### Involvement of Grsf1 in mitochondrial function

Previous studies have implicated GRSF1 in mitochondrial metabolism [11, 12, 26]. In our proteomic experiments indicated lower protein expression levels of constituents of the NADH: ubiquinone oxidoreductase complex (complex I) of the mitochondrial electron transport chain (Fig. 7C). Our in vivo data are consistent with the outcome of recent in vitro studies, in which the expression of functional GRSF1 protein was knocked-down in HEK293 cells using the RNAi technology [24]. In this study the authors reported that reduction of GRSF1 expression resulted in a marked down-regulation of the steady-state concentrations of constituents of complexes I and IV of the mitochondrial electron transport system [24]. On a functional level, the authors detected declining complex I activity after silencing of GRSF1 expression whereas complex IV activity was not significantly altered [24]. In humans, isolated complex I deficiency is the most common enzymatic defect of the oxidative phosphorylation disorders and clinically, patients present with a wide range of disorders ranging from lethal neonatal disease to adult-onset neurodegenerative disorders [40, 41]. There are a number of clinically relevant mutations of complex I proteins and our proteomic data indicated that the expression of some of them is differentially regulated in our systemic *Grsf1*^*−/−*^ mice (NDUFB9 [42], NDUFS2 [43], NDUFS6 [44], NDUFA6 [45], NDUFS1 [46], NDUFA10 [47], NDUFA8 [48], NDUFV2 [49], NDUFS4 [50], NDUFB10 [51]. Among these, the most widely studied protein is NDUSF4 [52, 53]. Mutations in the human *NDUSF4* gene cause the Leigh syndrome, a neurodegenerative disease with onset in infancy and childhood [54, 55]. Patients usually lack detectable NDUFS4 protein and exhibit complex I stability and/or assembly defects [56]. Interestingly, mice lacking a functional *Ndufs4* gene are characterized by growth retardation [57, 58]. Whether downregulated expression of complex I proteins are a suitable explanation for the observed decreased body weight gain of our systemic male *Grsf1*^*−/−*^ mice remains unclear for the moment. However, the *Grsf1*^*−/−*^ mice reported here may constitute a useful tool to explore this open question.

### GRSF1 and intracellular redox homeostasis

Since the GRSF1 protein regulates translation of the *Gpx4* mRNA it has been implicated in cellular redox homeostasis [8, 20]. GPX4 is a member of the glutathione peroxidase family, which comprises 8 different isoforms in humans [37]. These enzymes reduce organic and inorganic hydroperoxides to the corresponding alcohols employing glutathione as electron donor [59]. Owing to its structure [60] GPX4 does not only reduce simple organic and inorganic hydroperoxides but also more complex hydroperoxy ester lipids [61]. GRSF1 binds to an AGGGA motif in the 5ʹ-untranslated region of *Gpx4* mRNA [10] and upregulates translation of the transcript [20]. In an in vitro embryogenesis model RNAi mediated expression silencing of the *Grsf1* gene induced oxidative stress and led to impaired mid- and hindbrain development [62]. Both effects were reversed by transgenic overexpression of GPX4 [62]. Interestingly, in our proteome analyses GPX4-specific tryptic cleavage peptides were detected but overall GPX4 expression levels did not differ markedly between *Grsf1*^*−/−*^ mice and wildtype controls. We neither detected obvious developmental defects in the brain of *Grsf1*^−/−^ mice and this data challenge the results of our previous in vitro embryogenesis studies [20].

### Role of Grsf1 in erythropoiesis

In a previous study Grsf1 has been implicated in mouse erythropoiesis [19]. In this ex vivo study, the authors examined the impact of Grsf11 on murine erythroblasts development. They reported that Grsf1 binds to an AGGGGA sequence in the 5’ untranslated region of the *Use1* mRNA. In erythropoiesis, USE1 is required for the mTOR dependent amplification of the erythroid compartment in the bone marrow and therefore abrogation of Grsf1 expression in murine embryoblasts led to the formation of pyknotic red blood cell precursors. Based on this ex vivo data we expected impaired red blood cell parameters for *Grsf1*^*−/−*^ mice. This was, however, not the case. In fact, *Grsf1*^*−/−*^ mice carry a fully functional erythropoietic system, which is indicated by the lacking differences in the major erythropoietic parameters of *Grsf1*^*−/−*^ mice and wildtype controls (Fig. 5). There are a number of possible reasons for the obvious discrepancy between our in vivo results and the previous ex vivo experiments. One of these possibilities might be that a compromised GRSF1 signalling is compensated in our in vivo knock-out mice by the upregulation of potential compensatory systems. However, for the time being such compensatory mechanisms have not been identified but the *Grsf1*^−/−^ mice described here might be a useful model to identify such mechanisms.

### Role of Grsf1 in viral infections

GRSF1 has been implicated in host cell infections by influenza virus [21, 63], herpes simplex virus-1 [64] and human immunodeficiency virus 1 [65]. It also may play a role in papilloma virus induced cervical cancer [66]. Unfortunately, most of these conclusions are only supported by in vitro experiments but in vivo confirmation of these hypotheses is still pending. The systemic *Grsf1*^*−/−*^ mice described here may be useful to test them in different in vivo experimental strategies.

## Supplementary Information

**Additional file 1:** Raw RNA bulk sequencing data.

**Additional file 2:** RNA sequencing summary including differentially expressed genes and GO terms; Results from mass spectrometry including differentially expressed proteins and GO terms.

## Data Availability

All data generated or analysed during this study are included in this published article, its supplementary information files and publicly available repositories. The mass spectrometry proteomics data have been deposited to the ProteomeXchange Consortium via the PRIDE partner repository (https://www.ebi.ac.uk/pride/) with the dataset identifier PXD034916.
